# The prognostic value and economic benefits of coronary angiography-derived fractional flow reserve-guided strategy in patients with coronary artery disease

**DOI:** 10.1016/j.heliyon.2023.e17464

**Published:** 2023-06-22

**Authors:** Zhenzhou Zhang, Mengshi Xie, Xixi Dai, Zhiyong Duan, Zhiren Lu, Liangyin Cai, Rongrong Gu, Lei Shen, Zhong Xu, Weifeng Yao, Yunfei Liu, Minlei Liao, Hongyu Shi

**Affiliations:** a Department of Cardiology, Zhongshan Hospital Wusong Branch, Fudan University, 200094, China; b Medical Emergency Center of Baoshan District, Shanghai, 201901, China; c Department of Pharmacy, Wusong Hospital of Zhongshan Hospital, Fudan University, 200094, China

**Keywords:** Coronary angiography-derived fractional flow reserve, Coronary artery disease, Percutaneous coronary intervention, Coronary angiography, Prognostic level, Health economic benefits

## Abstract

**Objective:**

This study aims to investigate the prognostic value and economic benefit of coronary angiography-derived fractional flow reserve (caFFR) guided percutaneous coronary intervention (PCI) in patients with coronary artery disease.

**Methods:**

All patients with coronary artery disease (CAD) who underwent coronary angiography in our center between April 2021 and November 2021 were retrospectively enrolled and divided into the caFFR guidance group (n = 160) and angiography guidance group (n = 211). A threshold of caFFR≤0.8 was used for revascularization. Otherwise, delayed PCI was preferred. The patients were prospectively followed up by telephone or outpatient service at six months for major adverse cardiovascular events (MACE) of all-cause death, myocardial infarction or target vessel revascularization, stent thrombosis, and stroke. All in-hospital expenses were recorded, including initial hospitalization and re-hospitalization related to MACE.

**Results:**

There was no significant difference in the baseline characteristics between the two groups. There were 2 (1.2%) patients in the caFFR guidance group and 5 (2.4%) patients in the angiography guidance group with MACE events during the following six months. Compared with angiography guidance, caFFR guidance reduced the revascularization rate (63.7% vs. 84.4%, p = 0.000), the average length of stents implanted (0.52 ± 0.88 vs. 1.1 ± 1.4, *P* < 0.001). The cost of consumables in the caFFR guidance group was significantly lower than that in the angiography guidance group (33257 ± 19595 CNY vs. 38341 ± 16485 CNY, *P* < 0.05).

**Conclusion:**

Compared with coronary angiography guidance, caFFR guidance is of great significance in reducing revascularization and cost, which has significant health and economic benefits.

## 1 Introduction

Percutaneous coronary intervention (PCI) is one of the most commonly used treatment measures for significant coronary artery disease [1]. The landmark FAME [2], FAME 2 [3], and a series of subsequent studies [4,5] results established the status of FFR as the gold standard for ischemic assessment and its importance in the decision-making of PCI. However, the measurement of FFR usually requires extra pressure guide wires and vasodilators, which brings extra discomfort to patients, difficulty in operation, procedural time, and medical expenses, which hinders its large-scale clinical application and leads to the extremely low utilization rate of FFR [6].

Coronary Angiography-derived Fractional Flow Reserve (caFFR) is a new and practical technique to judge whether coronary artery stenosis is functional ischemia based on angiography in recent years, which can calculate the blood flow reserve fraction by using an optimized CAD algorithm without pressure guide wire and vasodilators. Compared with FFR, its accuracy and feasibility have been verified by a series of clinical trials [[7], [8], [9]]. However, it is unclear whether caFFR-guided PCI lesion selection can improve the prognosis compared with the standard angiographic guidance strategy in the real world. Therefore, a retrospective study comparing caFFR-guided revascularization and standard coronary angiography was conducted to evaluate the application value of caFFR-guided PCI treatment strategy for patients.

## 2 Objects and methods

### 2.1 Study patients

All patients underwent coronary angiogram in our single hospital, between April 2021 and November 2021, with at least one coronary vessel stenosis (defined visually at >50%) included in this retrospective study. Exclusion criteria included participants who had a myocardial infarction in the past six days; left ventricular ejection fraction (LVEF) ≤ 35%; estimated glomerular filtration rate <60 ml/min (or 1.73 m^2^), patients suffering from severe coagulation or hemorrhagic diseases; those who were allergic to iodine contrast media, or participated in or is participating in another clinical trial in the past month. Exclusion criteria for angiographic images included ostial lesions ≤3 mm away from aorta, total or subtotal occlusive lesion, insufficient contrast media, severe vascular overlap failing to expose the lesion location, deformation of target vessels, or poor angiographic image quality.

This study was approved by the Medical Ethics Committee of Wusong hospital of Zhongshan Hospital of Fudan University (No. 2021-SYY-10), and all patients signed informed consent forms.

### 2.2 Procedure and antiplatelet regimen

Percutaneous coronary angiography was performed following standard techniques. In the caFFR group, caFFR values were measured for all lesions in the vessels with a reference diameter of at least 2.5 mm. All lesions with caFFR ≤0.80 were treated with PCI, and lesions with caFFR >0.80 were treated with delayed PCI and optimized drug therapy.

The caFFR measurement method: All angiographic images and real-time aortic pressure waveform via caFFR special pressure sensor was transmitted to the coronary angiography blood flow reserve fraction measurement system (FlashAngio FFR System, Suzhou Runmaide Medical Technology Co., Ltd.). Two angiography images with fully exposed stenosis position and body position angle greater than 30 were selected to generate a simulated three-dimensional grid reconstruction structure of the coronary artery along the vascular path from coronary artery entrance to distal stenosis position. It was recommended that the length of the reconstructed blood vessel range from 60 mm to 80 mm. The TIMI frame counting method was used to obtain the blood velocity of the patient at rest, and FlashAngio measurement software used an optimized computational fluid dynamics algorithm to solve the Navistokes equation, analyze the hemodynamics of the reconstructed blood vessel, calculate the pressure difference between the proximal and distal ends of the stenosis, and then calculate the caFFR value of the target blood vessel segment.

In the control group, PCI or conservative therapy strategy was left to physician discretion. All patients who underwent PCI were pretreated with clopidogrel or ticagrelor and aspirin. Otherwise, a loading dose of 300 mg of clopidogrel or 180 mg ticagrelor and 300 mg aspirin was administered. Dual antiplatelet therapy was prescribed for at least 12 months, followed by aspirin (100 mg/d) indefinitely. Another optimal medical therapy was administered according to guidelines.

#### 2.2.1 Definitions and follow-up

A major adverse cardiac event (MACE) was defined as the composite endpoint of all-cause death, myocardial infarction, or target vessel revascularization and angina pectoris hospitalization. The cost in the hospital was recorded for cost-effectiveness analysis. Clinical follow-up was carried out by telephone or outpatient service one month and six months after discharge.

#### 2.2.2 Statistical analysis

Categorical variables were presented as frequencies (percentages)and were compared by likelihood ratio χ^2^ test or Fisher precision test. Continuous variables were presented as mean ± standard deviation, those with normal distribution were compared by a two-sample *t*-test, and the Wilcoxon rank sum test compared those with non-normal distribution. The Kaplan-Meier method was used to estimate the time-first event rate of each group, and the log-rank test was used to compare the data. All data were analyzed using SPSS 25.0.

## 3 Results

### 3.1 Baseline clinical characteristics

**Fig. 1:**
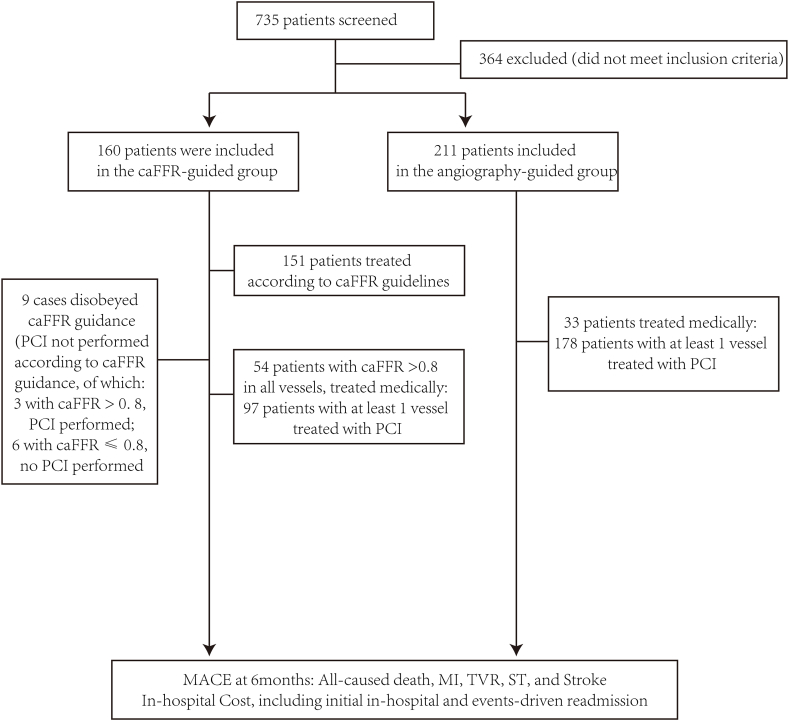
Flow chart of the study.

A total of 735 patients were screened, and 371 (50.48%) were selected, of which the angiography group guided 211 patients, and 160 patients were guided by the caFFR group (Fig. 1). The most common causes of screening failure are ostial lesions (n = 32), severe vascular overlap, target vascular deformation (n = 84), poor quality of angiographic images (n = 158), and no significant lesions detected (n = 77) .

The baseline characteristics were well balanced between the caFFR guidance group and the angiography guidance group (Table 1). Except for the P2Y12 inhibitors, the discharge medication of the two groups was similar.

**Table 1:** Baseline characteristics of the study population.

Item	caFFR guidance group (*n* = 160)	Angiography guidance group (*n* = 211)	*P*
Age, years old, mean ± standard deviation	67.5 ± 10.4	68.3 ± 10.8	0.439
Gender			
Male, *n* (%)	114 (71.3%)	145 (68.8%)	0.665
Female	46 (28.7%)	66 (31.2%)	0.783
Body mass index, kg/m^2^, mean ± standard deviation	24.9 ± 3.2	25.1 ± 3.4	0.862
Hypertension, *n* (%)	110 (68.8%)	144 (68.1%)	0.904
Hypercholesterolemia, *n* (%)	19 (11.9%)	26(12.5%)	0.864
Current smoking, *n* (%)	54 (33.8%)	67 (31.9%)	0.721
Diabetes mellitus, *n* (%)	67 (41.9%)	91 (43.1%)	0.821
Family history, *n* (%)	3 (1.9%)	5 (2.5%)	0.500^†^
History of myocardial infarction, *n* (%)	24 (15.0%)	38 (18.1%)	0.363
Allergy history, *n* (%)	14 (8.8%)	22(10.6%)	0.571
Left ventricular ejection fraction, %, mean ± standard deviation	0.61 ± 0.09	0.59 ± 0.09	0.245
Clinical manifestations			
Stable angina pectoris, *n* (%)	130 (81.3%)	164 (77.5%)	0.407
ST segment elevation myocardial infarction, *n* (%)	21 (13.1%)	30(14.4%)	0.745
Non-ST segment elevation myocardial infarction, *n* (%)	9 (5.6%)	17 (8.1%)	0.377
Discharge medication			
β-blocker, *n* (%)	142 (88.8%)	189 (89.6%)	0.800
Calcium antagonist, *n* (%)	53 (33.1%)	75 (35.5%)	0.627
Nitrate esters, *n* (%)	10 (6.3%)	14 (6.6%)	0.881
ACEI or ARB, *n* (%)	132 (82.5%)	176 (83.4%)	0.817
Statins, *n* (%)	158 (98.8%)	208 (98.6%)	0.887
Aspirin, *n* (%)	150 (93.8%)	202 (95.7%)	0.390
P2Y12 inhibitors, *n* (%)	119 (74.4%)	180 (85.3%)	0.008**

Note: * indicates that there is significant difference between caFFR group and angiography group (P < 0.05), * * indicates that there is extremely significant difference between caFFR group and angiography group (P < 0.01); † represents Fisher's exact test; caFFR: Coronary angiography-derived fractional flow reserve; PCI: Percutaneous coronary intervention; ACEI: Angiotensin converting enzyme inhibitor; ARB: Angiotensin II receptor blocker. The same below.

### 3.2 Procedural characteristics

As can be seen from Table 2, the average calculation time of caFFR was 4.13 ± 1.18 min per patient. In the caFFR guidance group, 102 patients underwent PCI with 113 vessels, and 58 were delayed with 84 vessels. The caFFR guidance group used fewer stents and contrast media on average.

**Table 2:** Angiographic characteristics of caFFR guidance group and angiography guidance group.

Characteristic	caFFR guidance group (n = 160)	Angiography guidance group (n = 211)	P
**PCI rate**, *n* (%)	102 (63.7%)	178 (84.4%)	0.000**
Drug-eluting stent, *n* (%)	87 (85.3%)	158 (88.8%)	0.398
Drug-eluting Balloon, *n* (%)	13 (12.7%)	16 (9.0%)	0.321
Plain balloon angioplasty, *n* (%)	2 (2.0%)	4 (2.2%)	1.000
**Number of diseased Vessel**			0.945
Single, *n* (%)	114 (71.2%)	147 (69.7%)	
Double, *n* (%)	39 (24.4%)	54 (25.6%)	
Triple, *n* (%)	7 (4.4%)	10 (4.4%)	
PCI rate per vessel, *n* (%)	113/213 (53.0%)	132/285 (81.4%)	0.000**
**Location of lesion**			0.107
Left main, *n* (%)	2 (0.9%)	4 (1.4%)	
Left anterior descending branch	121 (56.8%)	131 (46.0%)	
Left circumflex branch, *n* (%)	37 (17.4%)	56 (19.6%)	
Right coronary artery, *n* (%)	53 (24.9%)	94 (33.0%)	
**Stenosis rate in QCA**			0.797
30∼50 stenosis, *n* (%)	39 (18.3%)	53 (18.6%)	
50∼70 stenosis, *n* (%)	60 (28.2%)	86 (30.2%)	
70∼90 stenosis, *n* (%)	74 (34.7%)	102 (35.8%)	
Over 90%, *n* (%)	40 (18.8%)	44 (15.4%)	
Number of lesions per capita	1.3 ± 0.5	1.3 ± 0.5	0.920
Number of lesions treated per capita	0.7 ± 0.3	1.1 ± 0.3	0.000**
Average stent length	0.52 ± 0.88	1.1 ± 1.48	0.000**
Success rate of PCI lesions	211/213 (99.1%)	210/212 (98.6%)	0.387
Success rate of PCI operation	98/102 (96.1%)	139/147 (94.6%)	0.182
Contrast medium usage (mL)	161.0 ± 72.6	169.8 ± 73.3	0.000**
caFFR calculation time (min)	4.13 ± 1.18	/	/

**Fig. 2:**
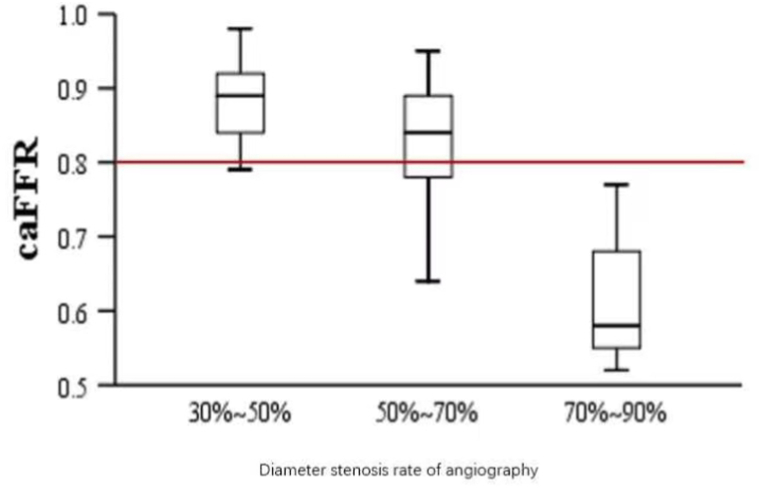
Angiographic severity and functional severity of coronary artery stenosis in the caFFR guidance group.

Among 213 lesions in 160 patients in the caFFR group, the percentage of diameter stenosis in quantitative coronary angiography (QCA) was 30%–50% in 39 cases, 50–70% in 60 cases, and 71–90% in 74 cases, and 91%–99% in 40 cases. Among all 213 lesions, 87 lesions were lower than the ischemic threshold (caFFR ≤0.8), 86 lesions were higher than the ischemic threshold (caFFR >0.8), and 40 lesions with diameter stenosis of 91%–99% underwent PCI without caFFR measurement. As shown in Table 3, when the QCA diameter stenosis of the lesion was 30%–50%, there was still one vessel with a caFFR value < 0.8, which was treated with a PCI strategy due to the physician's discretion. The average caFFR was 0.83 when the diameter stenosis was 50%–70%, 39 (65.0%) lesions had caFFR >0.8, 21 (35.0%) lesions had caFFR≤0.8 (Fig. 2). The average caFFR was 0.64 in 70%–90% stenosis, 9 lesions (12.2%) had caFFR >0.8, and 65 lesions (88.8%) had caFFR≤0.8 (Fig. 2).

**Table 3:** Lesion characteristics and caFFR value in caFFR guidance group.

	Diameter Stenosis in QCA		
	30%–50% (n = 39, 18.3%)	50%–70% (n = 60, 28.2%)	70%–90% (n = 74, 34.7%)
caFFR＞0.8	38	39	9
caFFR≤0.8	1	21	65
Mean caFFR of all blood vessels	0.89	0.83	0.64
caFFR >0.8 mean	0.89	0.87	0.84
caFFR ≤0.8 mean	0.79	0.73	0.62

### 3.3 Prognostic and cost analysis

The caFFR and angiography guidance groups were followed up for six months. The number of patients who dropped out in the caFFR guidance group and angiography guidance group was 1 and 2, respectively. 2 patients (1.3%) in the caFFR guidance group and four patients (2.5%) in the angiography guidance group had major composite endpoints within six months. Among them, one patient in the caFFR guidance group who did not follow caFFR guidance (caFFR <0.8, did not undergo PCI) underwent target vessel revascularization, as shown in Table 4.

**Table 4:** Clinical outcome of caFFR guidance and angiography guidance group in 6 months.

	caFFR guidance group (n = 160)	Angiography guidance group（n = 211）	P
Hospitalization (days)	7.5 ± 3.2	7.2 ± 3.1	0.547
MACE，n (%)	2 (1.2%)	5 (2.4%)	0.689
All-caused death *n* (%)	0	2 (0.9%)	0.604
Myocardial infarction, n (%)	0	0	
Target Vessel Revascularization, n (%)	1 (0.6%)	1 (0.5%)	1.000
Stent Thrombosis, *n* (%）	0	1 (0.5%)	1.000
stroke, n (%）	1 (0.6%)	1 (0.5%)	1.000

The economic evaluation of the two strategies mainly includes the initial hospitalization cost and the event treatment cost during the follow-up period. Initial hospitalization expenses include actual materials used and consumed medicines. The treatment cost of follow-up events includes the actual treatment cost due to MACE, as shown in Table 5. The total hospitalization cost of the caFFR guidance group was (58,258 ± 27,200 yuan), which was lower than that of the angiography guidance group 62,851 ± 22,2008 yuan. Among them, the cost of consumables in the caFFR guidance group was significantly lower than that in the angiography guidance group (33,257 ± 19,595 yuan vs. 38,341 ± 16,485 yuan, P < 0.05). During the follow-up period, the cost of MACE in the caFFR guidance group was also lower than that in the angiography guidance group (538 yuan 5609 yuan vs. 1620 yuan 11324 yuan), but there was no statistical difference.

**Table 5:** In-hospital cost of caFFR guidance group and angiography guidance group.

Item	caFFR guidance group (n = 160)	Angiography guidance group (n = 211)	P
Total initial hospitalization expenses (CNY)	58258 ± 27200	62851 ± 22008	0.098
consumables cost (CNY)	33257 ± 19595	38341 ± 16485	0.014*
Medicine cost (CNY)	4022 ± 2186	4154 ± 2190	0.590
Event cost during follow-up period (CNY)	538 ± 5609	1620 ± 11324	0.283
Total cost (CNY)	58896 ± 27186	64671 ± 21595	0.079

## 4 Discussion

In this single-center retrospective real-world study, compared with coronary angiography-guided PCI, caFFR guidance provided similar in-hospital and 6-month clinical outcomes with fewer PCI and reduced cost.

CaFFR is a new method for calculating blood flow reserve fraction based on coronary angiography in recent years, which is confirmed to have higher diagnostic accuracy (95.7%) in myocardial ischemia compared with guidewire FFR [10]. Similarly, Pulse's QFR [10,11], Cathworks' FFRangio [12], and Pie Medical's vFFR [13] are also FFR measurement products based on coronary angiography. In addition to contrast images, there are also FFR measurement products based on CT [14], IVUS [15], OCT [16], pressure microcatheter [17,18], etc.

It is the first study that demonstrated the value of caFFR in the decision-making of coronary artery disease in the real world. In this study, we found that of all the lesions with 50%–70% angiographic severity, only 35% lesions showed functional ischemia (caFFR ≤0.8), even in 70%–90% of lesions with angiographic stenosis severity, 12% lesions were no clinically significant, which is similar to the study of pressure wire FFR. Moreover, after six months of following-up, the MACE events rate was 1.2% in the caFFR-guidance group, which is lower than the angiography-guidance group.

Among the 213 target vessels in 160 patients of the caFFR group, 49 (30.6%) had a change in the treatment regimen. Specifically, 48 angiographic severely stenotic vessels (diameter stenosis rate was 50%–90%) were delayed after caFFR measurement (caFFR >0.8); PCI was performed on one vessel of a patient with hemodynamic obstructive lesions (caFFR <0.8), although there was no severe stenosis (diameter stenosis rate 30%–50% in QCA). The change of PCI strategy for angiographic severe stenosis lesions makes the caFFR guidance group treat fewer vessels and lesions than the angiography guidance group, thus greatly reducing the PCI rate and using fewer stents and contrast media on average. In 58 patients (36.3%), the PCI procedure was completely delayed because the caFFR values of all vessels were greater than 0.8, and long-term drug therapy alone was preferred, which explained why fewer patients used dual antiplatelet therapy during follow-up.

The cost of performing PCI in patients without functional ischemia increases [19]. A sub-group analysis of the FAME, FAME2 study showed that the FFR-guided PCI strategy resulted in significant cost savings by reducing stents, readmission, and MACE rates [20,21]. Although there was no significant difference in the total cost between the caFFR group and the contrast group during initial hospitalization and later follow-up, caFFR significantly reduced the cost of consumables. Under a centralized procurement policy in China, the cost of balloons, coronary stents, and medicine used in this study is extremely low. However, the cost of measuring caFFR is about 10,000 RMB, so the benefit of reduction of material and antiplatelet drugs used in the caFFR group is underestimated. However, reducing the PCI rate caused by a higher caFFR utilization rate will reduce the overall cost of PCI and bring higher comprehensive benefits. On the whole, caFFR guidance has higher significance in health economics.

As a result, caFFR can distinguish between lesions and blood vessels that require interventional therapy and those that can be safely delayed, lowering the incidence of early and late myocardial infarction. The caFFR-guided ischemia-driven revascularization was less than that of the angiography-guided group during the 6-month follow-up. Furthermore, caFFR guidance provided lower resource consumption, shorter operation time, reduced number of stents, less contrast agent use, and reduced radiation exposure for doctors and patients. It is similar to a previous study comparing FFR-based and angiography-based strategies [2,22]. However, caFFR is more easily adopted for angiography-based diagnostic and interventional procedures than for invasive physiological assessments. The caFFR strategy does not require specialized pressure guidewires and contrast media and can be easily repeated multiple times. With real-time preoperative, intraoperative, and postoperative assessments, caFFR provides additional benefits to both patient and operator with a much lower measurement time than guidewire FFR [23]. Actually, in this study, it took less than 5 min to complete the caFFR measurement. However, in the CorMicA trial, it took more than 60 min to complete the FFR measurement [23]. Consequently, caFFR contributes to the routine use of physiological assessment in clinical practice.

There were several limitations in this study. Firstly, this is a single retrospective center study with a limited sample size and only a 6-month following-up. However, it is the first study investigating the prognosis value and cost of caFFR in the real world. A larger, multi-center, randomized, prospective study should be conducted concerning the longer outcomes of caFFR guided strategy of coronary artery disease. Secondly, under the circumstance of the centralized procurement policy of China, the economic benefit of caFFR was underestimated. Nevertheless, the caFFR guidance strategy significantly reduced the usage of consumables related to PCI and consequential dual antiplatelet therapy, resulting in the conservation of health resources.

## 5 Conclusion

Compared to coronary angiography guidance, caFFR has a significant significance and role in evaluating the functional ischemia of coronary artery stenosis. The caFFR-guided strategy of selecting blood vessels and lesions reduced PCI rates, improved prognosis, and had significant health and economic effects.

## Production notes

### Author contribution statement

Zhenzhou Zhang: Performed the experiments; Analyzed and interpreted the data; Conceived and designed the experiments; Wrote the paper.

Mengshi Xie: Conceived and designed the experiments; Wrote the paper.

Xixi Dai, Zhiyong Duan, Zhiren Lu, Liangyin Cai, Rongrong Gu and Lei Shen: Performed the experiments; Analyzed and interpreted the data.

Zhong Xu, Weifeng Yao, Yunfei Liu and Minlei Liao: Analyzed and interpreted the data. Contributed reagents, materials, analysis tools or data.

Hongyu Shi: Conceived and designed the experiments; Wrote the paper.

### Data availability statement

Data will be made available on request.

### Additional information

No additional information is available for this paper.

## Funding statement

This study was supported by the Baoshan District Medical Key Discipline and Characteristic Brand Project (No. BSZK-2023-Z03).

## Declaration of competing interest

The authors declare that they have no known competing financial interests or personal relationships that could have appeared to influence the work reported in this paper.
